# The micro- and nanoscale spatial architecture of the seed mucilage—Comparative study of selected plant species

**DOI:** 10.1371/journal.pone.0200522

**Published:** 2018-07-24

**Authors:** Agnieszka Kreitschitz, Stanislav N. Gorb

**Affiliations:** 1 Department of Functional Morphology and Biomechanics, Kiel University, Kiel, Germany; 2 Department of Plant Developmental Biology, Institute of Experimental Biology, University of Wrocław, Wrocław, Poland; College of Agricultural Sciences, UNITED STATES

## Abstract

The seed coat mucilage envelope is formed just after hydration and surrounds the seed as a gel-like, transparent capsule. The mucilage envelope represents a special type of modified cell wall with all of the typical polysaccharides i.e. cellulose, pectins and hemicelluloses. The chemical composition of the mucilage is well-recognized but its structural organization remains unclear. In the presented study, we visualized the spatial architecture of the seed mucilage envelope of selected taxa which produce cellulose mucilage. Using critical point drying (CPD) and scanning electron microscopy (SEM) imaging, we demonstrated the structural details of the mucilage from the micro- down to the nanoscale. The mucilage, after CPD, had a visibly spatial structure which differed between the studied taxa; for example, a tangled organization in *Arabidopsis thaliana* and a more ordered arrangement in *Ocimum basilicum* were revealed. In general, the mucilaginous fibrillary components formed network made of long, unbranched, thicker cellulose fibrils together with shorter, thinner and, often branched other polysaccharides. Cellulose fibrils built a kind of scaffold for the rest of the components which were spread between them and/or covered their surface. The cellulose fibrils were attached to the seed surface, and therefore prevent the loss of the mucilage envelope during mechanical impacts. The loose architecture and special chemical composition of the mucilaginous cell wall is important for water binding and storage, which are crucial for the proper functioning of the seed mucilage envelope.

## Introduction

Mucilage production by seeds and/or fruits (diaspores) of many angiosperms is a widespread phenomenon known as myxodiaspory [1,2]. Mucilage is produced by mucilage secreting cells (MSCs) which are an integrative part of the seed/fruit coat. The formation of a gel-like envelope around the seed is caused by mucilage release as a result of hydration [3, 4]. The ability to form the mucilage is a plant’s adaptation especially to particularly dry or disturbed habitats or for plants with short life cycle. It plays important roles in germination regulation, in adhesion to the ground, or in promoting dispersal [5,6,7].

Due to the hygroscopic properties of mucilage, it absorbs and maintains the water around the seed and establishes favorable conditions for germination [8,9,10]. The capacity of mucilage imbibition depends on the amount of mucilage deposited in the MSCs. The increase of seed mass after hydration and mucilage formation can range e.g. from 3.22 times in *Linum usitatissimum* (with small amount of mucilage) [6] to 167 in *Alyssum minus* or even up to 589 times in *Artemisia sphaerocephala* (with thick mucilage layer) [11,12]. Mucilage envelope provides better contact of the seed with the soil and therefore also better water uptake in comparison to non-mucilaginous seeds [5]. It was observed that the presence of mucilage envelope is often associated with higher germination percentages, however, this effect can be also species-specific and influenced by some physical factors which affect the germination rate [11,13]. Mucilage layer can acts as a physical barrier for water excess and oxygen diffusion, and therefore preventing germination e.g. in unfavorable conditions [5,11,13].

As it was shown for *Arabidopsis thaliana* seed mucilage represents a specific modified secondary cell wall which is particularly rich in pectins [14]. Typically all plant cell walls share several common features. They are composed of three main polysaccharide groups i.e. cellulose microfibrils which form the mechanical framework for the matrix phase composed of pectins and hemicelluloses [15,16,17,18,19]. All of this components are also typically present in mucilage and were detected in mucilaginous diaspores of diverse plant taxa [4,20,21,22,23,24].

The mucilage layer is deposited in the apoplast of the MSCs during the differentiation of the seed [14,23]. It is generally accepted that the presence of cellulose fibrils in the envelope prevents the mucilage from being lost from the seed surface [20,21]. Cellulose fibrils play an important structural role in mucilage forming a type of scaffold in which other polysaccharides are entangled or cross-linked [20,24,25,26]. Such structural interactions between polysaccharides were suggested for the mucilaginous cell wall from biochemical analyses. The detected hemicelluloses were associated with the cellulose, which means that their role in the mucilage is similar to their role in the primary cell wall. In addition, branched chains of pectins can presumably interact with the cellulose microfibrils [20,25,26,27,28]. However, in spite of the fact that the cell wall composition is well characterized, the detailed spatial-3D arrangement of polysaccharides within the mucilaginous cell wall has not been revealed. The spatial organization of the cell wall components has not been reproduced directly in details which demonstrate the shape, size and distribution of the polysaccharides.

It can be hypothesized that the specific mucilage composition and spatial architecture might be connected to particular habitat. For example the presence of thick and long cellulose threads could be beneficial for plants inhabiting dry, open areas e.g. arid, semi-arid or steppes. The fibrils could help in better anchorage to the ground and prevent against further dispersal by the wind. Such an example could be seeds of *Neopallasia pectinata*, inhabiting steppes in China and Mongolia, with heterogenous mucilage architecture [24]. This species produces huge amount of mucilage with very long cellulose threads which play also important structural role as a scaffold for other components [24].

Mucilage envelope due to its adhesive properties can interact with different surfaces. After hydration to the mucilage envelope can adhere the soil particles increasing the contact area and also fixing in this way the diaspore to the ground. Another important interaction is the adherence to animals’ body (e.g. birds) what allow for the diaspores dispersal [5].

The specific chemical composition, high imbibition potential and good adhesive and frictional properties of mucilage envelope are important features that are partially utilized in food technology and medicine. Mucilage is used e.g. as a binding agent or release retardant in the tablet formulations, as an emulsifying agent in hydrogels and as a lubricant [29]. Many mucilaginous diaspores are also very popular as food additives: e.g. *Salvia hispanica* (Chia) seeds are used in juices [30]. Different recipes and uses of the whole mucilage diaspores of *Linum usitatissimum*, *Ocimum basilicum*, *Salvia hispanica* are widely known and can be found in Internet.

The mucilaginous cell wall structure is mostly recognized on biochemical, genetic and some microstructural analyses [17,18,23,24,31,32,33]. They often applied various invasive techniques such as enzymatic or chemical treatments, which can destroy and/or influence cell wall organization and its individual components. This may also cause the loss of important information concerning the cell wall organization. Additionally, they can only detect chosen components of the wall [15,31,34]. Among microscopic techniques, a high resolution analysis including an Atomic Force Microscope (AFM), Transmission and Scanning Electron Microscopes (TEM, SEM), and Cryo-SEM were used to visualize the cell wall architecture [15,32,35,36]. However, they also mostly use invasive preparation methods and there is therefore still no single imaging technique which has resulted in obtaining structural information reproducing the quasi-native state of the mucilage.

The chemical composition of mucilage envelope has been intensively studied but the direct imagining of spatial arrangement of its components is still not presented. Therefore the main aim of the present study was to visualize the mucilaginous cell wall spatial architecture using less-invasive preparation techniques, which allowed us preservation of the material in its almost native state. For our studies comparative studies we selected taxa representing different genera which diaspores produce an abundant mucilage envelope. The main question asked was how this gel-like structure is maintained during the hydrated phase. We also wanted to visualize: (1) how the mucilage envelope is structurally organized after hydration and air-drying as well as after critical point drying; (2) how the mucilaginous cell wall components are spatially arranged; (3) what the differences between the micro- and nanostructure of the mucilage between different plant taxa are.

## Material and methods

### Material

Seeds of following taxa were used: (*Brassicaceae*) *Arabidopsis thaliana* L. (Sadków, Lower Silesia, Poland, 2011, leg. et det. Magdalena Turzańska, University of Wrocław, Poland), *Artemisia annua* L. (Sweet wormwood, *Asteraceae*) (Wrocław, Poland leg. et det. A. Kreitschitz), *Artemisia leucodes* (*Asteraceae*) (obtained from the collection of Prof. Joan Vallès, Barcelona University, Catalonia, Spain) *Lepidium sativum* (Garden cress, *Brassicaceae*) L. (from commercial supplier: CNOS-GARDEN, Poland, 2014), *Ocimum basilicum* L. (Basil, *Lamiaceae*) (from commercial supplier: TORSEED, Toruń, Poland, 2014), *Salvia sclarea* L. (Clary sage, *Lamiaceae*) (leg. A. Kreitschitz, collection from the Botanical Garden, University of Wrocław, Poland, 2014). For the analysis, only matured seeds were used. For each experiment at least three seeds of each taxon were examined.

These taxa were selected for this study due to the ability of their seeds to produce a copious mucilage envelope. Also these seeds easy accessible from either commercial suppliers or from wild populations. There are some morphological, anatomical, biochemical and/or tribological studies of seed mucilage of selected taxa (e.g. *Lepidium sativum*, *Ocimum basilicum*, *Artemisia* ssp., *Arabidopsis thaliana*) in the literature, but detailed ultrastructural analysis is still lacking. Therefore the present study might be an important supplement extending the existing knowledge.

### Mucilage determination

For the determination of the basic mucilage morphology and the main components i.e. pectins and cellulose, seeds were hydrated in water and then stained with two main dyes: seeds were hydrated in water and then stained with ruthenium red (0.1%, w/v, Merck) for the pectins [37] and for cellulose with safranin (1% w/v) and with Direct Red 23 (0.1% w/v, Sigma-Aldrich) for specific cellulose staining [38,39]. To detect the starch in the mucilage of *Ocimum basilicum* we used the solution of potassium iodide with iodine in water [38]. The images were taken using an Olympus BX-50 light microscope connected to a DP71 camera with Cell B imaging software (Olympus BX50, Olympus Optical Co., Poland) and Zeiss CLSM microscope (LSM 700 AXIO ZEISS; excitation LP 5550, emission 560 nm).

### General morphology of the seed coat mucilage air-dried after hydration

Round cover slips were attached to the SEM stubs using double-sided adhesive conductive tape containing carbon. The seeds were hydrated for 30 min and individually put on the cover slips and air-dried for 24 h. The seeds of studied taxa were coated with gold palladium and visualized in a SEM (Hitachi S-4800, Hitachi High-Tech. Corp., Tokyo, Japan.

### Spatial architecture of the seed coat mucilage after hydration and CPD-drying

Seeds of studied plant species were hydrated for 30 min to obtain a mucilage envelope and were then dehydrated in an ascending ethanol series from 10% to 100% and dried using a critical point dryer (Typ E 3000, United Kingdom, Quorum Technologies Ltd, New Haven, East Sussex, England), mounted on the SEM-stubs and coated with gold palladium. After coating, the seeds were immediately visualized in the SEM (Hitachi S-4800, Hitachi High-Tech. Corp., Tokyo, Japan).

The width measurements of fibrillarly material was done for studied taxa (Table 1). We do not present the statistical analysis because of small and different counts of measurements. The material was very delicate and unstable under the electron beam and therefore it was difficult to obtain high magnification pictures and to do more measurements.

**Table 1:** Width measurements of fibrillary material of studied taxa.

Taxon	Main chains; mean; range [nm]	Count of measurements	Cross-links; mean; range [nm]	Count of measurements
*Arabidopsis thaliana*	20.8 (±3.1); 16.7–27.08	10	11.6 (±1.5); 10.1–14.2	16
*Lepidium sativum*	22.7 (±2.4); 19.7–28.3	30	15.5 (±2.0); 11.6–18.8	30
*Ocimum basilicum*	57.3 (±22.9);20.7–127.4	43	16.3 (±2.7); 11.3–23.6	38
*Salvia hispanica*	32.7 (±6.0); 24.7–44.2	30	18.4 (±2.4); 14.1–23.8	43
*Artemisia annua*	29.6 (±6.5); 19.7–49.4	28	15.2 (±1.8); 11.5–18.6	21
*Artemisia leucodes*	70.0 (±17.0); 32.1–116.2	25	14.9 (±2.5); 10.5–19.8	30

## Results

### Mucilage identification

Based on the staining reactions two main components of mucilage were identified. Ruthenium red revealed pectins which were stained pink/red (Figures A-F in S1 File). They formed the main mass of the mucilage envelope. Staining with safranin for cellulose was positive for all of studied taxa (Figures A´-F´ in S1 File). Additionally for cellulose detection we used a specific dye—Direct Red 23, which clearly distinguished the cellulose skeleton in a form of delicate, straight threads stretching around the seed (Figures A-H in S2 File). Based on the staining reactions the mucilage of all studied taxa can be classified as cellulose type.

**Fig 1:**
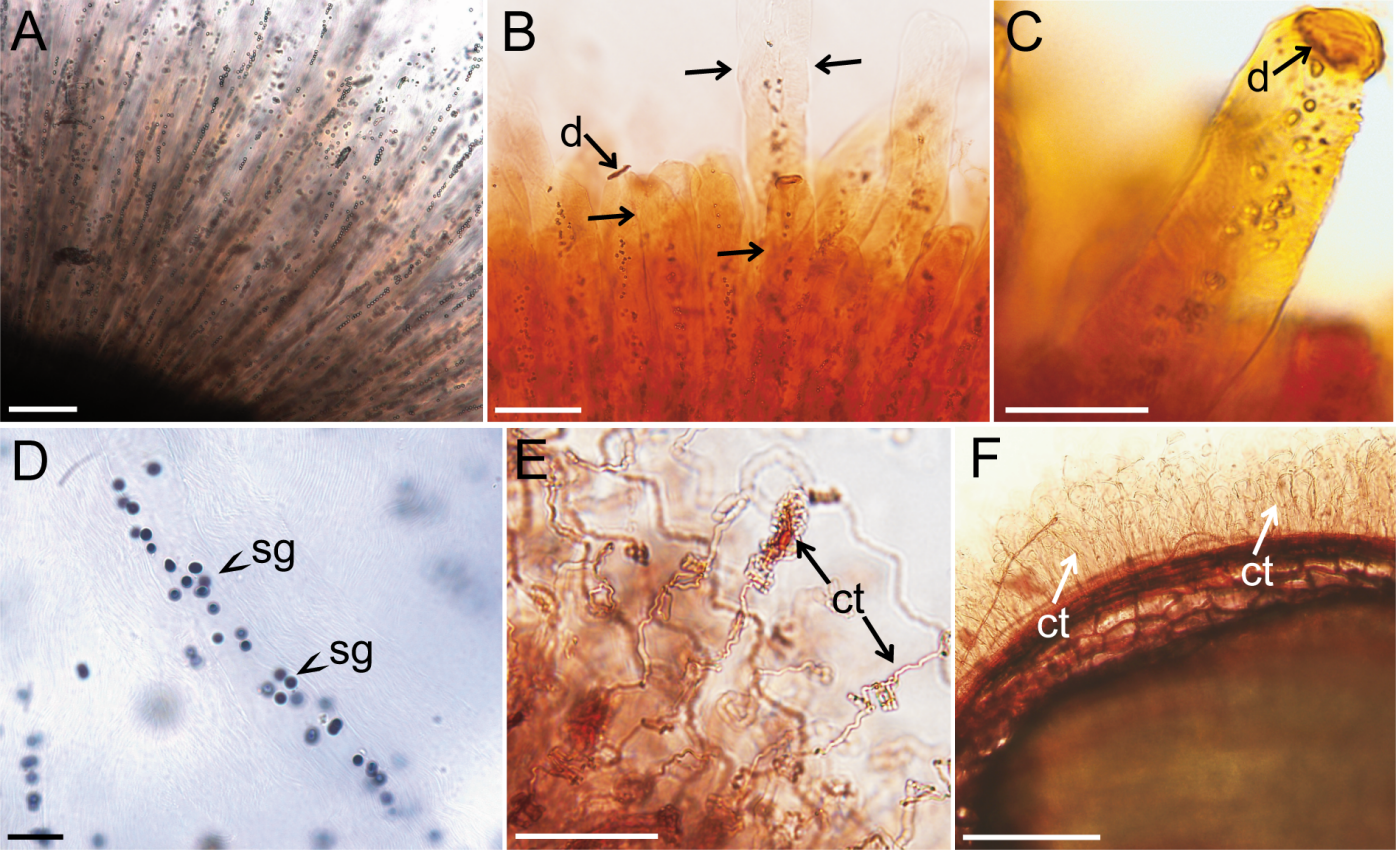
Mucilage morphology. A-D *Ocimum basilicum* mucilage envelope. A. Unstained mucilage with visible small granules-starch grains; B-C. Staining with safranin revealed ‘tubule’-like structure of mucilage (arrows) with spirally-coiled cellulose fibrils which are closed on the top with the disc-like structure (arrow); D. *Ocimum basilicum*—dark color indicates starch grains stained with I in KI; E *Salvia sclarea* stained with safranin. Visible thick, partially uncoiled wavy, cellulose threads; F. *Artemisia annua* mucilage envelope stained with safranin with observable cellulose threads. **Abbreviations**: ct–cellulose threads, d—disc, sg–starch grains **Scale bars:** A, B– 100 μm, C, E– 50 μm, D– 20 μm, F– 200 μm.

Staining reactions demonstrated specific morphology of mucilage envelope in *Ocimum basilicum*. The envelope was formed by individual tubule-like structures with visible spirally-coiled cellulose threads (Figure D, E in S2 File, Fig 1B and 1C) and small granules (Fig 1A–1D). The staining reaction with I in KI allowed to identify them as starch grains (Fig 1D). On the top of each ‘tubule’, a type of disc-like structure was observed (Fig 1B and 1C), to which the cellulose threads were attached. The disc prevented this ‘tubular’ structure from opening and threads uncoiling. Staining with safranin shown wavy, partially coiled form of cellulose threads in *Salvia sclarea* mucilage (Fig 1E). The cellulose threads of the rest of studied taxa were delicate, long and stretched around the seed forming a radial skeleton in the mass of pectins (Figure A, B, G, H in S2 File, Fig 1F).

### The morphology of the dried seed coat mucilage

**Fig 2:**
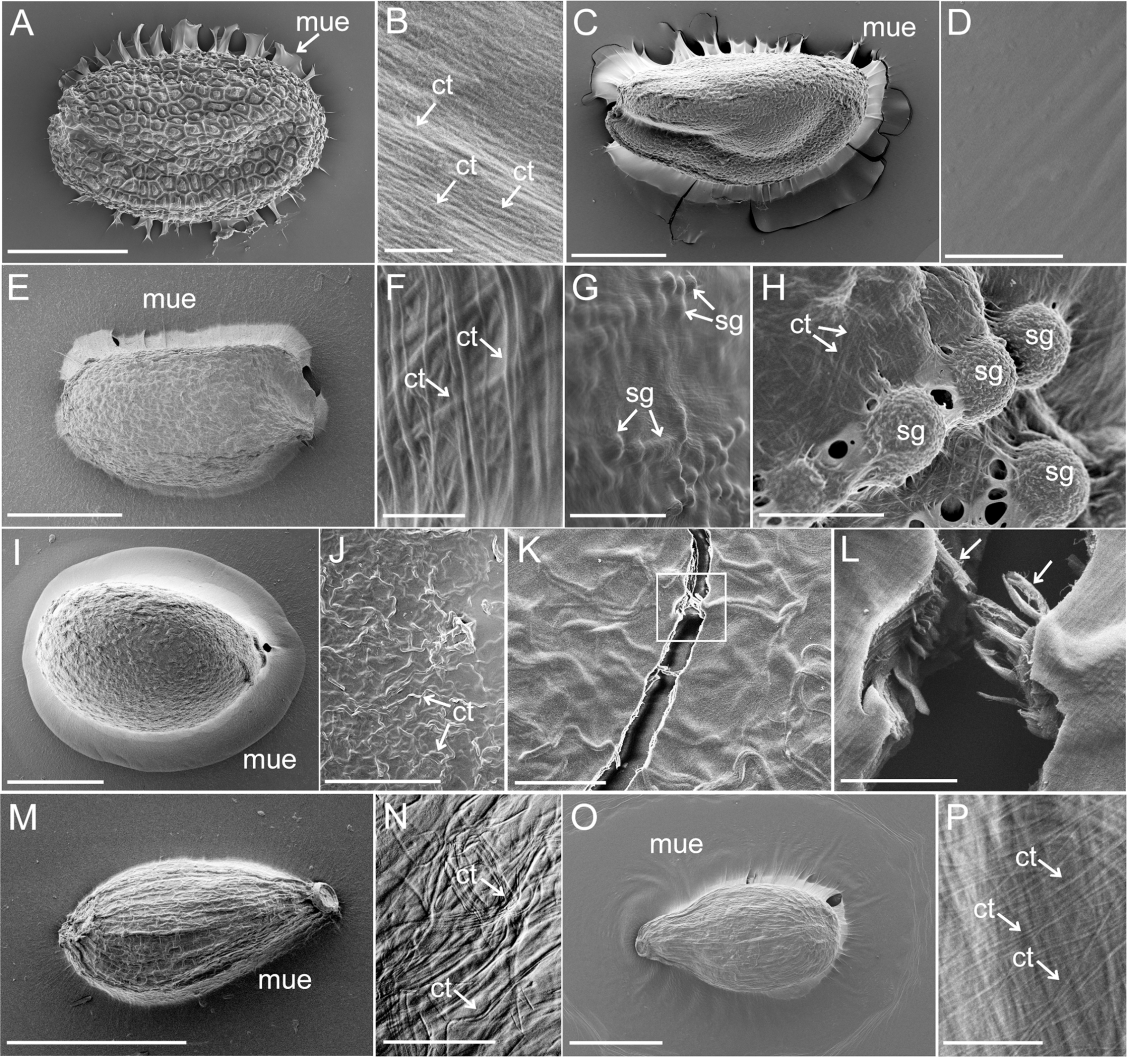
The morphology of the air-dried seed coat mucilage. A-B. *Arabidopsis thaliana*. A. Characteristic mucilage sheets (arrow) spread between the seed and substratum (glass); B. The mucilage surface at higher magnification with the visible cellulose threads (arrows) running parallel; C-D. *Lepidium sativum*. C. Seed attached with mucilage to the substratum; D. Smooth surface of mucilage envelope. E-H. *Ocimum basilicum*. E. Seed with the mucilage envelope; F. The mucilage surface with cellulose threads (arrows); G-H. Starch grains visible under mucilage envelope; I-L. *Salvia sclarea*. I. Seed with mucilage envelope; J. Visible fragment of mucilage envelope with wavy cellulose threads (arrows); K. Broken fragment of mucilage envelope; L. Magnification of K (box) showing the broken cellulose threads (arrows) imbed in the mucilage; M-N. *Artemisia annua*. M. A very delicate mucilage around the seed; N. Cellulose threads (arrows) running parallel or in different directions within the mucilage; O-P. *Artemisia leucodes*. Abundant mucilage envelope around the seed with visible cellulose threads (arrows); **Abbreviations**: ct–cellulose threads, mue–mucilage envelope, sg–starch grains **Scale bars:** A—200 μm, F, M– 500 μm, C, E, I, O– 1 mm, D, K– 10 μm, B, F– 500 nm, G– 20 μm, H– 5 μm, J– 50 μm, L– 2 μm, N—30 μm, P– 1 μm.

After hydration, the mucilage had expanded around the seed and during the drying process, the water loss caused that the mucilage envelope collapsed and adhered to the glass surface (Fig 2A, 2C, 2E, 2I, 2M and 2O). After complete desiccation, the mucilage envelope was visible as a transparent layer spread on the glass around the seed.

The mucilage surface of *Lepidium sativum* was almost smooth and homogenous (Fig 2C and 2D). In case of other taxa cellulose threads were observable as imbed in the dried mucilage envelope. They were randomly arranged as running parallel or in different directions (Fig 2B, 2F, 2H, 2J, 2K, 2N and 2P).

In *Ocimum basilicum*, the mucilage covered round starch grains spread as protrusions under the mucilaginous layer (Fig 2G and 2H). Also thick cellulose threads were good recognized (Fig 2F and 2H). The wavy cellulose threads of *Salvia sclarea* were also visible in the dried mucilage envelope (Fig 2J and 2K). On the mucilage break (cross-section) they can be also well recognized (Fig 2L).

### Mucilage spatial architecture visualized in SEM after hydration and CPD-drying

**Fig 3:**
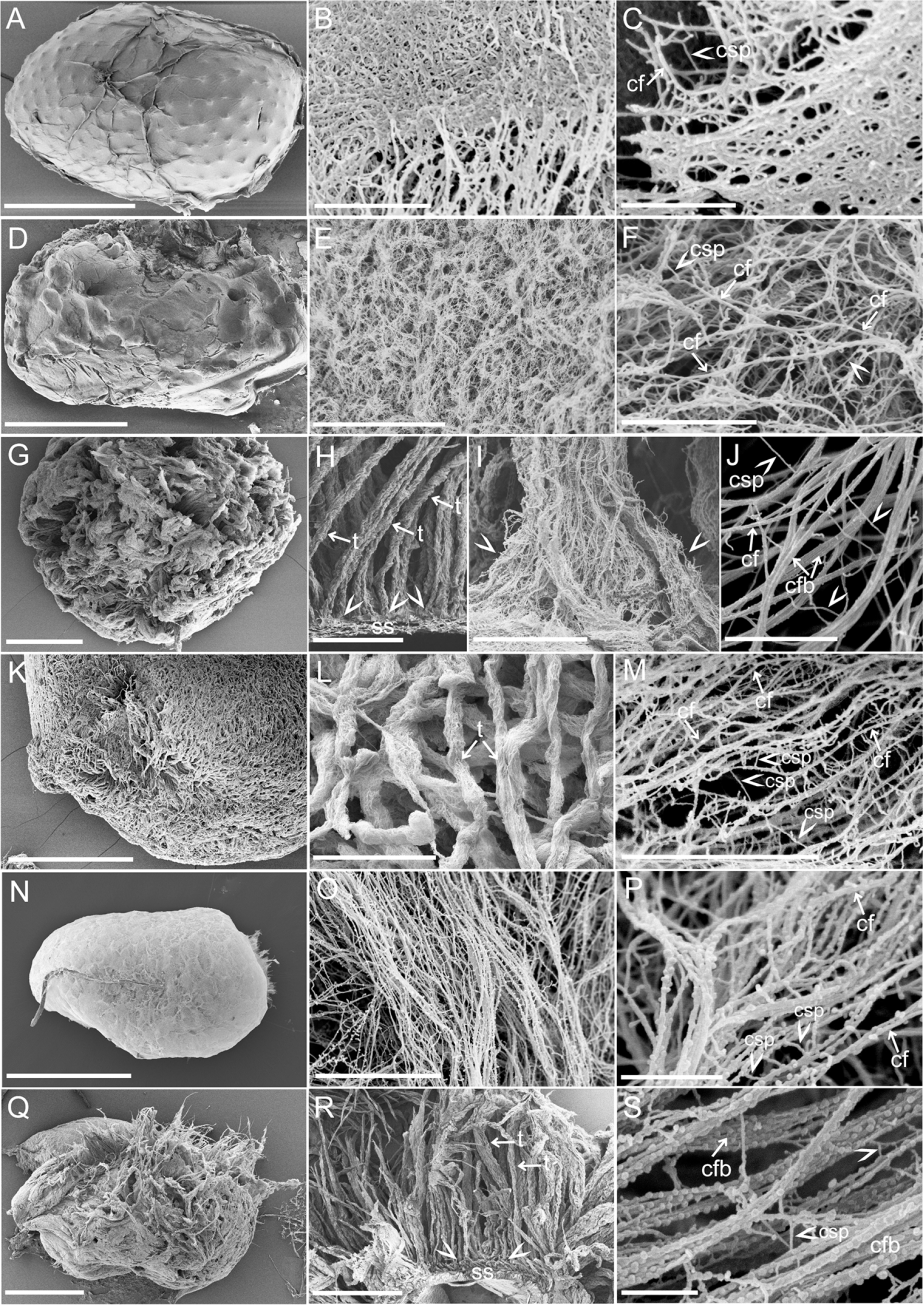
Mucilage spatial architecture after hydration and CPD-drying visualized in SEM. A-C. *Arabidopsis thaliana*. A. The seed surface tightly covered with the mucilage; B. Mucilage components organized in a tightly-tangled net-like structure; C. Thicker cellulose fibrils (arrow) cross-linked by shorter chains (arrowhead); D-F. *Lepidium sativum*. D. The abundant mucilage envelope; E. Delicate fibrils form a densely-organized mucilage envelope; F. The main, unbranched cellulose fibrils (arrows) cross-linked by shorter chains (arrowhead). G-J. *Ocimum basilicum*. G. Mucilage forms an uneven envelope densely-covering the seed surface; H. Mucilage material organized in ‘tubules’ (arrows) attached to the seed surface (remainders of the cell wall of mucilage secreting cells); I. The site where the cellulose fibrils are attached to the seed surface, the ‘base of the tubule’; J. Cellulose fibrils (arrows) with other components (cross-linking polysaccharides) (arrowheads) spread between the fibrils; K-M. *Salvia sclarea*. K. The surface of the seed is covered with dense mucilage layer; L. The ‘tubules’ (arrows) structure of the mucilage envelope at higher magnification; M. The net-like structure at the site, where the cellulose fibrils constitute the main scaffold and where they are cross-linked by other components (matrix polysaccharides; arrowheads); N-P. *Artemisia annua*. N. Very delicate mucilage envelope; O. Parallel organization of the cellulose fibrils; P. Net-like structure of the mucilage with visible cellulose fibrils (arrows), granules covering their surface and crosslinking components (arrow heads); Q-S. *Artemisia leucodes*. Q. Very abundant mucilage envelope around the seed; R. Organization of the mucilage components in ‘tubules’ attached to the seed surface (arrows); S. The cellulose fibrils (arrows) very tightly covered with granules and cross-linked by shorter chains (arrowheads). **Abbreviations**: cf–cellulose fibrils, csp–crosslinking polysaccharides, cfb–cellulose fibril bundles, t–‘tubules’, ss–seed surface **Scale bars**: A, N, R– 500 μm, B, F, J– 1 μm, C, P, S– 500 nm, D, G, K, Q– 1 mm, E, I– 5 μm, H, L– 100 μm, M, O– 2 μm, K– 5 μm.

Critical Point Drying (CPD) permitted the removal of water from the mucilage envelope and the preservation of the mucilage architecture. The mucilage did not collapse as in previous experiments but showed distinct, spatial organization (Fig 3). That the mucilage components formed a thick network on the seed surface was visible soon after CPD. The detailed visualizations, under diverse magnifications of the SEM and measurements (Table 1) demonstrated various micro- and nanoscale features of the mucilage envelope in individual plant taxa.

The mucilage of studied taxa had a visible spatial structure apart of *Arabidopsis thaliana* seeds which revealed more compressed organization of the mucilaginous material (Fig 3A). The mucilage envelope revealed the presence of long and short fibrils arranged in a net-like spatial structure in all taxa (Fig 3B, 3C, 3E, 3F, 3J, 3M, 3O, 3P and 3S). After hydration and cell rupture, the fibrils stayed attached to the remaining cell wall of the MSCs, so the mucilage envelope was retained on the seed surface (Fig 3H, 3I and 3R). The main, thicker fibrils present in the mucilage envelope represent a kind of scaffold for the rest of the components i.e. short, linear or branched fibrils spread between (Fig 3C, 3F, 3J, 3M, 3P and 3S). Interestingly the fibrillary material of *Arabidopsis thaliana* and *Lepidium sativum* mucilage envelope was more homogenous in the size as in the rest of the studied taxa (Table 1). In *Ocimum basilicum*, *Salvia sclarea* and both *Artemisia* species the main thicker fibrils and thinner chains spread between them were better distinguishable (Table 1, Fig 3J, 3M, 3P and 3S).

The very irregular arrangement of the fibrillary material was characteristic of *Arabidopsis thaliana* and *Lepidium sativum* (Fig 3B, 3C, 3E and 3F). The fibrils were not differed in their size very clearly. The mucilage of *Arabidopsis thaliana* (Fig 3B and 3C) had a more compact form in compare to *L*. *sativum* which was more loosely organized (Fig 3E and 3F).

Mucilage of *Ocimum basilicum* and *Salvia sclarea* had a very characteristic organization of components in the form of distinct, long ‘tubules’ (Fig 3H and 3L) distributed very tightly at the seed surface. The mucilage components within the envelope of both *Ocimum basilicum* and *Salvia sclarea* were arranged as long, unbranched fibrils parallel and with short, thinner, branched chains connecting them (Fig 3J and 3M). In some places, the starch grains were also visible within the mucilage envelope of *Ocimum basilicum*.

The mucilage architecture of *Artemisia annua* and *Artemisia leucodes* was similarly organized. The main fibrils running parallel were visible as individual fibrils or aggregations forming bundles (Fig 3P and 3S). Interestingly in *Artemisia leucodes*, such ‘tubules’ (Fig 3R) like in *Ocimum basilicum* were also visible on the cross-sections. The mucilage envelope of *Artemisia annua* was more delicate however the long chains and shorter, spread between them were also detectable (Table 1, Fig 3O and 3P).

The surface of the main, thick fibrils was smooth, resembling that in e.g. *Arabidopsis thaliana* (Fig 3C), *Lepidium sativum* (Fig 3F) or rough, covered with small granules, like e.g. in *Ocimum basilicum* (Fig 3J), *Salvia sclarea* (Fig 3M), *Artemisia annua* (Fig 3P), and *Artemisia leucodes* (Fig 3S).

## Discussion

In the presented study, we revealed the spatial architecture of the mucilage envelope, as well as demonstrated the structural appearance i.e. size, shape and organization of its components for the first time in the quasi-native state. The micro- and nanoarchitecture of the mucilaginous cell wall was visualized using a less-invasive sample preparation: Critical Point Drying (CPD) and Scanning Electron Microscopy (SEM). It allowed us to preserve the intact spatial architecture of the mucilage and to reveal details, which cannot be detected using e.g. traditional light microscopy staining or immunolocalization procedures.

The form of seed coat mucilage varies depending on the amount of water absorbed. Immediately after hydration, the mucilage presents a gel-like capsule (Figures A-F, A´-F´ in S1 File) which changes after complete drying out in a delicate, transparent, thin layer and sticks to the substrate. In our study, we demonstrated how different methods of water removal i.e. air-drying or CPD revealed mucilage structure. The air-dried mucilage changes its form into a thin, flat, transparent layer (Fig 2). In contrast, the CPD method allowed the preservation and visualization of the spatial organization of the mucilage (Fig 3). Due to the comparison of the results obtained with both methods used, we demonstrated the distribution of components within the mucilage, their shape and structure. The mucilage components are organized in a specific spatial net-like architecture.

### Cellulose fibrils constitute the main scaffold in the mucilage envelope

The seed coat mucilage represents a pectin-rich, modified secondary cell wall. All of the main components, typical of the cell wall, are present in the mucilaginous envelope [3,4,14, 23,24,40]. The cellulose represents a linear, unbranched homopolymer of β-1,4-linked glucose residues [41,42].

The cellulose was detected after staining reactions (safranin, Direct Red) and visible as long threads. At this level of observation (light microscope) we cannot distinguish individual cellulose fibrils therefore, we also used SEM images to demonstrate the fibrilar structure of the cellulose. The long, unbranched fibrils, stretching radially from the seed surface, perfectlyy correspond to the typical features of cellulose fibrils. Our SEM images demonstrated that they are anchored to the remainders of the primary cell wall of mucilage-secreting cells (Fig 3H, 3I and 3R). This observation correspond to our previous studies on the mucilage of *Neopallasia pectinata* [18]. This fibrillary scaffold can prevent the mucilage envelope against separation from the seed and maintain the stability of the mucilage form. The cellulose fibrils are responsible for the retention of polysaccharides (pectins and hemicelluloses) within the gel-like capsule and around the seed [26,40].

### Matrix polysaccharides spread between the cellulose fibrils

Short, (un)branched cross-links between the cellulose fibrils represent most possibly the matrix polysaccharides. The mucilage envelope of seeds from the families *Asteraceae*, *Brassicaceae*, *Lamiaceae*, i.e. the families to which the studied taxa belong, revealed this can possess of diverse pectins, e.g. homogalacturonan, branched rhamnogalacturonan I, and hemicelluloses e.g.: xyloglucan, highly branched xylan, galactomannan and arabinoxylan [21,23,25,43]. Many of those polysaccharides are characterized by the presence of side chains which are responsible for cross linking the fibrils and presumably contribute to the mechanical stability of the mucilaginous cell wall structure [26]. The hemicelluloses are responsible for maintaining the cellulose architecture as well as pectins’ anchorage to the seed surface. Such a role of hemicellulose was described in the *Arabidopsis thaliana* mucilage envelope [26]. Pectins, due to their ability to bind water, determine the water holding capacity of the mucilage [6,7]. Therefore, they constitute important component of the mucilage envelope, which serves as water reservoir for the diaspore. Our visualization method directly revealed this complex organization of the mucilaginous components and interactions between them, which results in this net-like architecture formation. The spatial structure of mucilaginous cell wall differs from the typical cell wall by the loose arrangement of its components that results from the main function of mucilage, which is water accumulation.

### Width of the mucilage envelope components

Based on width measurements on SEM visualizations (Table 1), we can describe cellulose as microfibrils and microfibril bundles. They were visible as long, unbranched fibrils, clearly thicker than the cross-links spread between them. Their mean size varied from 20.8 nm (*Artemisia thaliana*) to 70 nm (*Artemisia leucodes*). The size of cellulose microfibrils strongly differs dependent on e.g. the taxon and/or cell wall composition [33,36,41,44]. The estimated average width of cellulose microfibrils gained by spectroscopic methods was 30 nm [45] and corresponds to our results (Table 1). However, in case of e.g. *Ocimum basilicum* (Fig 3J) and *Artemisia leucodes* (Fig 3S) thick bundles of cellulose microfibrils were also visible. The results are comparable to our previous results [24].

The mean width of shorter (un)branched chains, spread between the main thicker fibrils, was comparable in all of the taxa examined (Table 1), and was consistently smaller than the mean width of cellulose microfibrils. These chains can correspond to the matrix polysaccharides, i.e. pectins and hemicelluloses, present in the mucilage envelope. Their size is comparable to the results obtained for *Neopallasia pectinata* mucilage envelope [24]. Similar cross-linking fibrils have already been observed by other authors, e.g. in the primary cell wall or in ginkgo tracheid cell wall, and were regarded as hemicelluloses [15,33,44].

### Mucilage envelope architecture variability as an expression of its chemical composition

The CPD+SEM method also demonstrated the structural diversity of the mucilage envelope in seeds of plants belonging to different taxa. Such differences in mucilage architecture might result from variations in chemical composition and/or structure of individual components e.g. the presence of linear or branched chains of polysaccharides, structure of cellulose fibrils. The mucilage composition demonstrates the presence of the main components that can vary between taxa [23].We can therefore suppose that this could be one of the main reasons for the variation in mucilage architecture. Such influence of the mucilage chemical composition on e.g. its physical characters was demonstrated on *Linum usitatissimum* and *Plantago lanceolata* mucilaginous seeds [6,7].

The physical properties of cellulose and its chemical behavior/reactivity are strongly influenced by the specific arrangement of the cellulose molecules [46]. The cellulose microfibril size, structural organization and interactions with other elements (polysaccharides) present in the mucilage can influence the whole mucilage envelope structure. We observed a very delicate net-like structure in *Arabidopsis thaliana* and *Lepidium sativum*, where the mucilage components (cellulose fibrils and matrix polysaccharides) did not differ distinctively. Thicker cellulose fibrils were typical of e.g. *Ocimum basilicum*, *Salvia sclarea* and *Artemisia leucodes* showed a characteristic tubular morphology of mucilage. However, this organization can disappear later after hydration.

The identity of the small granules covering the fibrils of e.g. *Artemisia annua* or *Artemisia leucodes* remains unclear. We suppose that they can represent e.g. some proteins which have been found recently in the seed mucilage of *Arabidopsis thaliana*. Different proteins are naturally elements of the cell wall and play diverse roles [47]. Our previous studies demonstrated the presence of xylan in the mucilage of *Neopallasia pectinata*. Xylan was spread in form of segments in parallel and most likely interacting with the cellulose fibrils [24]. Previous studies showed that cellulose microfibrils as well as the elementary fibrils can be covered by hemicelluloses [48]. Thus, we suggest that the granular structures can also represent e.g. xylan molecules interacting with cellulose fibrils.

### Methodology of the mucilage structure visualization

The chemical composition and morphological organization of the mucilage has previously been studied in detail by many authors [23], but the spatial architecture of the mucilage remained only hypothetical. Experiments with hydrated and air-dried seeds revealed seed mucilage which formed a thin film with some wrinkles and drapes ‘gluing’ the seed to the surface [43,49]. Some attempts were made to visualize the mucilage morphology of *Arabidopsis thaliana* seeds using Cryo-SEM and CPD+SEM for nutlets of *Salvia hispanica* L. [43,49,50,51], however, these studies only demonstrated general appearance of the mucilage envelope.

In our high-magnification images we presented arrangement of polysaccharides in the mucilage envelope. The shape, size and spatial organization of them could be observed at the nanoscale down to the most precise detail(s). The nanoscale images of *Salvia hispanica* cellulose mucilage were presented using AFM [43]. The mucilage structure of this species is formed by elongated microfibrils (mucilage fibres) organized in a network. However, the AFM only demonstrated the surface topography of the samples, whereas our CPD+SEM method revealed a spatial architecture of the mucilage (Fig 3).

The combination of CPD and SEM was also applied for anatomical studies of some myxospermic diaspores from *Asteraceae*, *Lamiaceae*, and *Brassicaceae* [52]. However, in this case, the results only revealed a very basic morphology of the mucilage. The visualization demonstrated only general features such as spiral or uncoiled mucilage strands on the seed surface, mucilage filaments or long coiled or uncoiled hairs [52]. Our studies with the application of the CPD+SEM technique to the seed mucilage seems to be a very promising approach for structural studies of the mucilaginous cell wall of seeds of other plant taxa.

The differences in the mucilage spatial architecture might be important in the pharmaceutical industry, where the seed mucilage is used in pharmaceutical formulations (tablets, gels, membranes, lubricants) [29]. Also post-production analysis of the spatial architecture of e.g. mucilage based hydrogels, lubricants or films might be of importance, to prove appropriate modifications of the mucilage according to the expected properties.

## Conclusions

In the presented study, we revealed the spatial architecture of the mucilage envelope, as well as demonstrated the structural appearance i.e. size, shape and organization of its components for the first time. The mucilage envelope had a net-like structure differing between the plant taxa studied. The cellulose fibrils constituted a kind of scaffold for the rest of mucilage components. This specific, loose architecture of the mucilaginous cell wall demonstrates its adaptation potential to accumulation of a great amount of water. Remarkable branched nature of the mucilage components can also be responsible for the establishing of larger contact area with the substrate (soil, animal body) by means of different physico-chemical mechanisms. The previously unknown architecture of the mucilaginous cell wall was visualized using a less-invasive sample preparation: critical point drying (CPD) and scanning electron microscopy (SEM). It allowed us to preserve the intact spatial architecture of the mucilage and to reveal micro- and nanostructural details which cannot be detected using traditional light microscopy staining and immunlocalizations procedures. The application of the CPD+SEM technique to the seed mucilage seems to be a very promising approach for structural studies of the mucilaginous cell wall of seeds other plant taxa from such genera as e.g. *Salvia* and *Plantago*. Diaspores of *Arabidopsis thaliana* and its diverse lines might be an interesting candidate to study the changes in the mucilage architecture caused by different mutations. The results of the present structural study could also be helpful for the interpretation of biomechanical properties (adhesion, friction) of the mucilaginous cell wall. The CPD+SEM method can be potentially used in the studies of industrial products based on the seed mucilage (hydrogels, lubricants).

## Supporting information

S1 File Detection of pectins and cellulose in the mucilage envelope. A-F ruthenium red staining reveals the presence of pectins building the main mass of mucilage. A’-F’ safranin stains cellulose fibrils embedded in the mass of pectins; A, A’—*Arabidopsis thaliana*, B, B’–*Lepidium sativum*; C, C’–*Ocimum basilicum*; D, D’–*Salvia sclarea*; E, E’–*Artemisia annua*; F, F’–*Artemisia leucodes*. **Scale bars:** A, E, A’, E’– 200 μm, B-D, B’-D’, F’– 1000 μm, F– 500 μm.
(TIFF)

S2 File Detection of cellulose in the mucilage envelope. A-H. Staining with Direct Red revealed the presence of cellulose in the mucilage. A. *Arabidopsis thaliana*; B. *Lepidium sativum*; C-E. *Ocimum basilicum*, D. Characteristic ‘tubules’ in the mucilage; E. Magnification of the ‘tubule’ with visibly spirally coiled cellulose threads; F. *Salvia sclarea*; G. *Artemisia annua*; H. *Artemisia leucodes*. **Scale bars:** A-C, F, H– 200 μm, D-E– 100 μm, G—50 μm.
(TIFF)
