# Supplementary figures and images for: The micro- and nanoscale spatial architecture of the seed mucilage—Comparative study of selected plant species
Source: PLoS One. 2018 Jul 24;13(7):e0200522. doi: 10.1371/journal.pone.0200522 (PMC6057646; doi:10.1371/journal.pone.0200522)

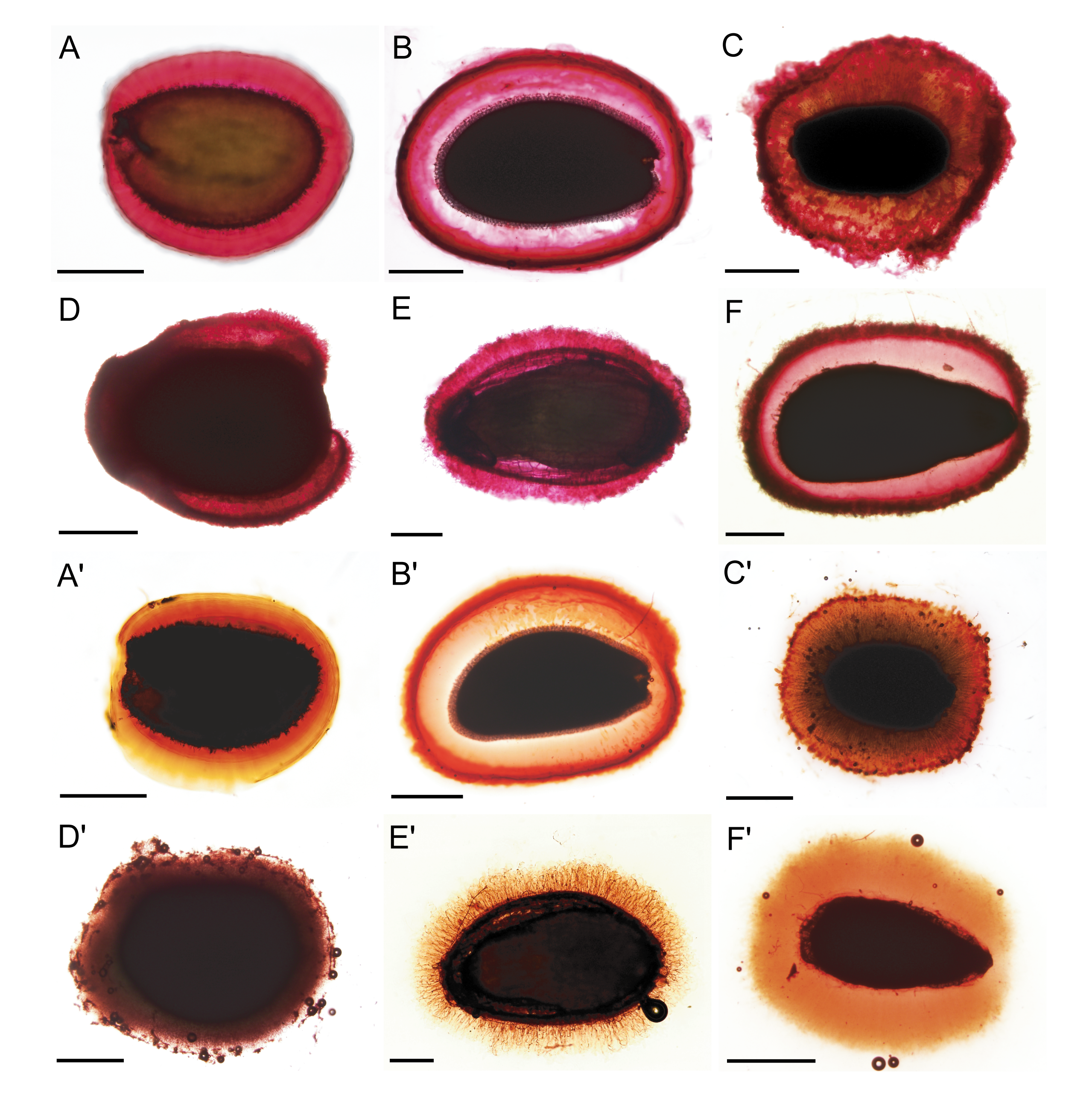

Supplement: S1 File — A-F ruthenium red staining reveals the presence of pectins building the main mass of mucilage. A’-F’ safranin stains cellulose fibrils embedded in the mass of pectins; A, A’—Arabidopsis thaliana, B, B’–Lepidium sativum; C, C’–Ocimum basilicum; D, D’–Salvia sclarea; E, E’–Artemisia annua; F, F’–Artemisia leucodes. Scale bars: A, E, A’, E’– 200 μm, B-D, B’-D’, F’– 1000 μm, F– 500 μm. (TIFF) [file pone.0200522.s001.tiff]

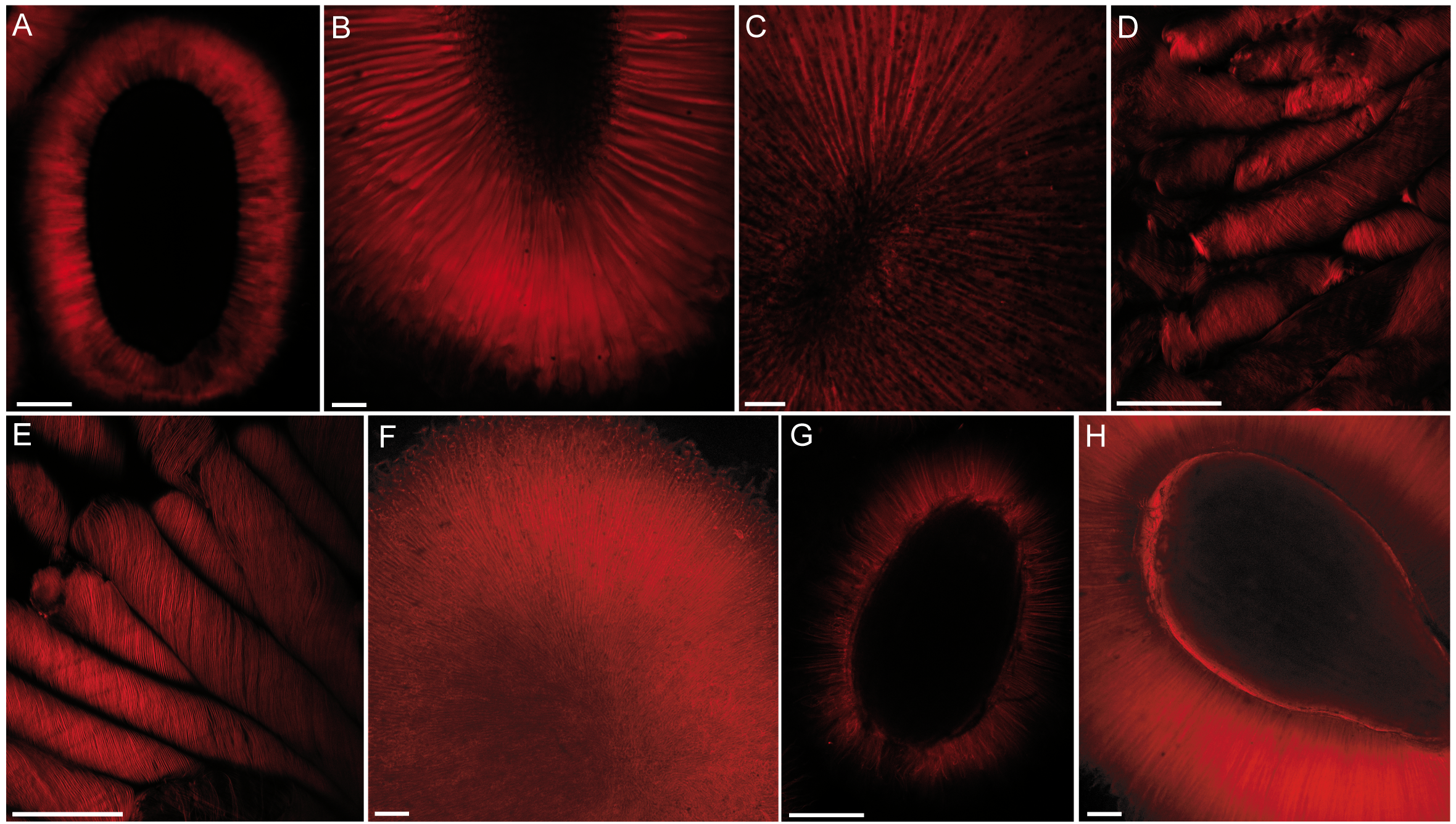

Supplement: S2 File — A-H. Staining with Direct Red revealed the presence of cellulose in the mucilage. A. Arabidopsis thaliana; B. Lepidium sativum; C-E. Ocimum basilicum, D. Characteristic ‘tubules’ in the mucilage; E. Magnification of the ‘tubule’ with visibly spirally coiled cellulose threads; F. Salvia sclarea; G. Artemisia annua; H. Artemisia leucodes. Scale bars: A-C, F, H– 200 μm, D-E– 100 μm, G—50 μm. (TIFF) [file pone.0200522.s002.tiff]
